# dsRNA Binding Domain of PKR Is Proteolytically Released by Enterovirus A71 to Facilitate Viral Replication

**DOI:** 10.3389/fcimb.2017.00284

**Published:** 2017-06-28

**Authors:** Yu-Hsiu Chang, Kean Seng Lau, Rei-Lin Kuo, Jim-Tong Horng

**Affiliations:** ^1^Graduate Institute of Biomedical Sciences, College of Medicine, Chang Gung UniversityTaoyuan, Taiwan; ^2^National Defense Medical Center, Institute of Preventive MedicineTaipei, Taiwan; ^3^Department of Biochemistry and Molecular Biology, College of Medicine, Chang Gung UniversityTaoyuan, Taiwan; ^4^Research Center for Emerging Viral Infections, College of Medicine, Chang Gung UniversityTaoyuan, Taiwan; ^5^Molecular Infectious Disease Research Center, Chang Gung Memorial HospitalTaoyuan, Taiwan; ^6^Research Center for Chinese Herbal Medicine and Research Center for Food and Cosmetic Safety, College of Human Ecology, Chang Gung University of Science and TechnologyTaoyuan, Taiwan

**Keywords:** EV-A71, 3C protease, PKR, Enterovirus, interaction effects

## Abstract

Enterovirus 71 (EV-A71) causes hand, foot and mouth disease in young children and infants, but can also cause severe neurological complications or even death. The double-stranded RNA (dsRNA)-dependent protein kinase R (PKR), an interferon-induced antiviral protein, phosphorylates the regulatory α-subunit of the eukaryotic translation initiation factor 2 in response to viral infection, thereby blocking the translation of cellular and viral mRNA and promoting apoptosis. The cleavage of PKR after infection with poliovirus, a prototype enterovirus, has been reported by others, but the underlying mechanism of this cleavage and its role in viral replication remain unclear. In the present study, we show that viral 3C protease cleaves PKR at a site, Q188, which differs from the site cleaved during apoptosis, D251. In contrast to the conventional phosphorylation of PKR by dsRNA, EV-A71 3C physically interacts with PKR to mediate the phosphorylation of PKR; this effect is dependent on 3C protease activity. Overexpression of a catalytically inactive PKR mutant (K296H) accelerates viral protein accumulation and increases virus titer, whereas a K64E substitution in the dsRNA binding site abolishes this advantage. We also demonstrate that PKR cleavage mediated by EV-A71 3C protease produces a short N-terminal PKR fragment that can enhance EV-A71 replication, in terms of viral RNA, viral protein, and viral titers. We conclude that PKR is co-opted by EV-A71 via viral protease 3C-mediated proteolytic activation to facilitate viral replication.

## Introduction

Enterovirus A71 (EV-A71), which belongs to the *Enterovirus* genus of the *Picornaviridae* family, is the major pathogen of human hand, foot, and mouth disease (HFMD). Severe infection with EV-A71 may lead to various neurological complications and even death in some patients, especially children under 5 years old (Lin et al., 1998). EV-A71 is a small, non-enveloped, positive-sense, single-stranded RNA virus. The viral genome is ~7,500 nucleotides in length, with a single open reading frame that encodes a large precursor polyprotein. This precursor is proteolytically processed into mature structural and non-structural proteins. The structural proteins (VP4, VP2, VP3, and VP1) constitute the capsid shell and the non-structural proteins (2A, 2B, 2C, 2BC, 3A, 3B, 3AB, 3C, 3D, and 3CD) are associated with viral replication (McMinn, 2002). In addition to its activity in viral protein processing, 3C protease is linked to a number of biological processes. When expressed in neuronal cells, 3C protease induces apoptosis through caspase activation (Li et al., 2002). In addition, the polyadenylation factor CstF-64 has been identified as a substrate for 3C protease. Cleavage of CsF-64 by 3C protease impairs host RNA processing and adenylation, thereby providing a suitable environment for viral replication (Weng et al., 2009). Moreover, 3C protease blocks type I interferon (IFN) responses by targeting innate immune factors: it inhibits interferon regulatory factor (IRF) 3 activation (Lei et al., 2010) and cleaves TIR-domain-containing adapter-inducing interferon-β (TRIF; Lei et al., 2011), IRF7 (Lei et al., 2013), and IRF9 (Hung et al., 2011), which mediate antiviral and immunoregulatory activities. 3C protease also mediates the cleavage of the transforming growth factor β-activated kinase 1 (TAK1)/TAK1/MAP3K7 binding protein (TAB)1/TAB2/TAB3 complex to interfere with nuclear factor (NF)-κB activation (Lei et al., 2014). A recent report showed that 3C protease interacts with and cleaves NLR family pyrin domain containing 3 (NLRP3) to counteract the protective role of the NLRP3 inflammasome against EV-A71 infection (Wang et al., 2015).

IFN-induced double-stranded RNA (dsRNA)-activated protein kinase R (PKR) is an IFN-stimulated gene (Gale and Katze, 1998; Peters et al., 2001; Pindel and Sadler, 2011) and acts as a pathogen recognition receptor (Gilfoy and Mason, 2007) by recognizing dsRNA, a typical by-product of viral infection, for IFN induction. PKR consists of two functionally distinct domains: an N-terminal regulatory domain and a C-terminal catalytic kinase domain. The regulatory domain contains two dsRNA-binding motifs; binding of dsRNA induces PKR dimerization and allows the exposure of the catalytic site, autophosphorylation, and activation of the kinase (Wu and Kaufman, 1997; Nanduri et al., 2000; Dar et al., 2005; Dey et al., 2005). Activated PKR catalyzes the phosphorylation of the regulatory α-subunit of the eukaryotic translation initiation factor 2 (eIF2α; Meurs et al., 1992; Clemens and Elia, 1997), consequently blocking the initiation of mRNA translation, which results in the global arrest of both cellular and viral protein synthesis and can lead to apoptosis in response to virus infection (Balachandran et al., 1998).

In addition to, its role in translational control through eIF2α phosphorylation, PKR plays a role in regulating several signal transduction cascades in the cell. The transcription factor nuclear factor NF-κB can be activated indirectly by PKR, independent of its kinase function, via association with tumor necrosis factor receptor-associated factor (TRAF) and activation of the IκB kinase complex (Gil et al., 2004; Bonnet et al., 2006). PKR has also been shown to play a role in the activation of p38 mitogen-activated protein kinase (MAPK) and the stress-activated protein kinase (SAPK)/c-Jun amino-terminal kinase (JNK; Goh et al., 2000), which are also key components in the host innate immune response. Furthermore, recent studies have revealed a kinase-independent role for PKR in apoptosis-associated speck-like protein assembly and caspase-1 activation during inflammasome formation (Hett et al., 2013).

Viruses have developed many mechanisms to counteract the antiviral function of PKR. Influenza virus NS1 (Chien et al., 2004), rotavirus NSP3 (Langland et al., 1994), and vaccinia E3L protein (Langland and Jacobs, 2004) bind to and sequester viral dsRNA, thus avoiding PKR activation. Adenovirus, Epstein-Barr virus, and hepatitis C virus produce RNA with secondary structures that bind directly to PKR but do not lead to the activation of the kinase (Langland et al., 2006). The Japanese encephalitis virus blocks PKR-mediated growth repression via NS2A (Tu et al., 2012). The interaction of hepatitis C virus NS5A and E2 with PKR directly inhibits PKR dimerization and activation (Gale et al., 1998; Taylor et al., 1999). PKR is rapidly degraded in poliovirus (PV)-infected cells (Black et al., 1989). PV is a prototype member of the *Picornaviridae* family, and studies have demonstrated that although PV infection induces PKR and eIF2α phosphorylation, there is significant degradation of PKR after PV infection. It has been suggested that the destabilization of PKR in PV-infected cells is caused by cooperation between viral dsRNA and a cellular protease, representing an example of viral-counteraction strategies. However, the detailed mechanism of PKR degradation remains unclear. In this study, we observed the cleavage of PKR during EV-A71 infection. This effect required 3C protease, which mediated PKR cleavage independently of caspases. Furthermore, we demonstrate that the kinase activity of PKR plays a negative role in EV-A71 infection. Our results also indicate that the 3C protease-generated fragments of PKR facilitated viral replication.

## Materials and methods

### Cell culture, virus, and infection

Human rhabdomyosarcoma (RD) and human embryonic kidney (HEK) 293T cells were cultured in Dulbecco's modified Eagle's medium (DMEM, Invitrogen, Carlsbad, CA, USA) that contained 10% heat-inactivated fetal bovine serum (Gibco BRL, San Francisco, CA, USA) at 37°C in humidified air with 5% CO_2_. The EV-A71 (TW/2331/98, genotype C) virus strain was produced from an infectious clone generously provided by Dr. Mei-Shang Ho (Academia Sinica, Taiwan). EV-A71 propagation and titer determination by plaque-forming assay using RD cells were performed essentially as described previously (Chang et al., 2012). Viral infection was carried out by preadsorbing EV-A71 at a multiplicity of infection (m.o.i.) of 10 for 1 h [1 to 0 h post-infection (p.i.)] at 37°C, and the unbound virus was removed and washed with phosphate-buffered saline (PBS). The cells were then cultured in DMEM supplemented with 2% fetal bovine serum and harvested at the indicated times p.i.

### Plasmids and constructions

Full-length PKR was amplified by PCR from cDNA prepared from RD cells and the product was inserted into the *Nhe*I and *Pst*I sites of pLKO_AS3Wpuro-eGFP-C1, pLKO_AS3Wpuro-nHAcFlag and pLKO_AS3Wpuro vectors, which were obtained from the National RNAi Core Facility (Academia Sinica, Taiwan). PKR variants K296H, D251N, K64E, A67E, Q138A, Q150A, Q154A, Q163A, Q188A, and PKR(1–188)K64E were constructed by site-directed mutagenesis using Pfu DNA polymerase (Stratagene, La Jolla, CA, USA). To construct plasmids P1 (encoding a precursor of viral capsid proteins VP4, VP2, VP3, and VP1), P2 (encoding a precursor of viral proteins 2A, 2B, and 2C), and P3 (encoding a precursor of viral proteins 3A, 3B, 3C, and 3D), fragments and viral proteases 2A and 3C of EV-A71 (TW/2331/98) cDNA were cloned into the *Nhe*I and *Eco*RI sites of the pLKO_AS3Wpuro-cFlag vector. The H40D, C147S, and R84Q mutants of 3C were produced using a site-directed mutagenesis kit (Stratagene). All constructs were confirmed by subsequent sequencing. Plasmids were transfected into designated cells using Lipofectamine 2000 (Invitrogen) as described by the manufacturer.

### Antibodies and reagents

Rabbit polyclonal antibody against EV-A71 3A was prepared as described (Tang et al., 2007). Mouse monoclonal antibodies against glyceraldehyde 3-phosphate dehydrogenase (GAPDH) (G8795) and Flag (F1804) were purchased from Sigma-Aldrich (St Louis, MO, USA). Rabbit monoclonal antibodies against phospho-eIF2α (#3597) and PKR (Ab32052, against amino acids 50–150 of human PKR) were obtained from Cell Signaling (Beverly, MA, USA) and Abcam (Cambridge, UK), respectively. Mouse monoclonal antibody against green fluorescent protein (GFP) (SC-9996) and rabbit polyclonal antibodies against eIF2α (SC-11386) and poly (ADP-ribose) polymerase (PARP) (SC-7150) were obtained from Santa Cruz Biotechnology (Santa Cruz, CA, USA). Mouse monoclonal antibody against EV-A71 3C and rabbit polyclonal antibody against phospho-PKR were obtained from GeneTex (Irvine, CA, USA). Poly(I:C) and 2-aminopurine (2-AP) was purchased from Invivogen (San Diego, CA, USA). Staurosporine was purchased from Sigma-Aldrich.

### Western blot analysis

The protein concentration was measured with a protein assay kit (Bio-Rad, Richmond, CA, USA). Equal amounts of whole-cell extracts were separated by 8–12% sodium dodecyl sulfate-polyacrylamide gel electrophoresis (SDS–PAGE). After electrophoresis, proteins were transferred to a polyvinylidene difluoride (PVDF) membrane (Millipore, Billerica, MA, USA). The membranes were blocked for 1 h at room temperature in 5% dried milk and then were probed with the indicated primary antibodies at an appropriate dilution overnight at 4°C. The following day, the membranes were incubated with horseradish peroxidase (HRP)-conjugated anti-mouse or HRP-conjugated anti-rabbit antibody. HRP was detected using Immobilon Western HRP Chemiluminescence Substrate (Millipore).

### Flow cytometry for apoptosis detection

RD cells in 6-well plates were transfected with 3C and 3C H40D/C147S mutant and treated with 2-AP. Forty-eight hours later, apoptosis was measured by flow cytometry using an Annexin V Kit (SC-4252 AK, Santa Cruz Biotechnology) according to the manufacturer's protocol. Briefly, the cells were dissociated with trypsin, washed with PBS twice and then stained with Annexin V-fluorescein isothiocyanate (FITC) and propidium iodide (PI) for 15 min. After staining, cells were analyzed using a CytoFLEX flow cytometer and Kaluza software (Beckman Coulter, Brea, CA, USA).

### Reverse transcription-quantitative PCR (RT-qPCR)

Total RNA extraction and reverse transcription were performed using an RNeasy Mini Kit (Qiagen, Venlo, Netherlands) and Superscript cDNA synthesis kit, respectively. Total RNA was converted into cDNA using random primers. The synthesized cDNA was measured by qPCR (Applied Biosystems® 7500 fast Real-Time PCR System) using a QuantiTect SYBR Green PCR kit (Qiagen) with the following gene-specific primers. EV71, CCC CTG AAT GCG GCT AAT C and GAT TGT CAC CAT AAG CAG C; GAPDH, ATC CTG GGC TAC ACT GAG CA; and GGT CCA GGG GTC TTA CT. Human GAPDH gene expression was used as an internal control. All qPCR experiments were performed in triplicate using samples with no template as a negative control.

### Immunoprecipitation

HEK-293T cells (7 × 10^6^ cells/well, 6-well plate) were transfected with 2 μg of various plasmids using Lipofectamine 2000, and were harvested 24 h post-transfection for further experiments. For the assay, transfected cells were washed twice with PBS and then scraped into a lysis buffer (150 mM NaCl, 1% Triton X-100, 0.5% sodium deoxycholate, 50 mM Tris-HCl, pH 7.5, and 2 mM EDTA) with freshly added protease inhibitors. Lysates of cells were incubated with anti-Flag antibody at 4°C overnight on a rotator in the presence of protein A/G agarose beads (Roche, Basel, Switzerland). After incubation, beads were washed three times, and the precipitated proteins were boiled at 95°C in SDS-PAGE sample buffer. Samples were resolved by SDS-PAGE and were transferred to a PVDF membrane for western blot analysis.

### Statistics

A student *t*-test was used for two-tailed comparisons. A *P* < 0.05 was considered significant. The symbols * and ** were used to indicate *p* < 0.05 and < 0.01, respectively.

## Results

### PKR is phosphorylated and cleaved during EV-A71 infection

**Figure 1:**
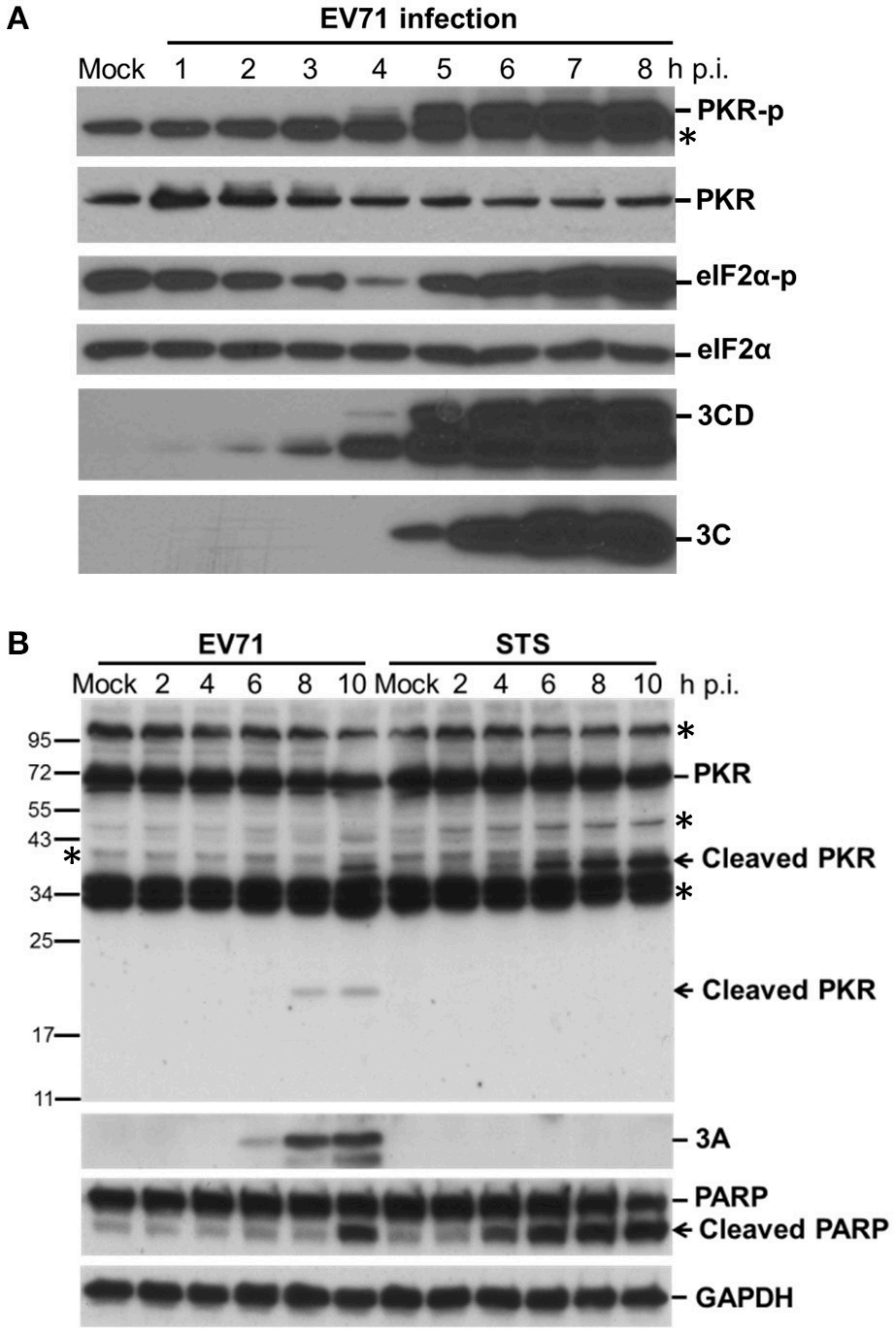
EV-A71 infection results in the phosphorylation and cleavage of PKR. **(A)** Time course of cellular protein phosphorylation and protein levels after EV-A71 infection. RD cells were infected with EV-A71 at an m.o.i. of 10. At the indicated times post-infection, cell extracts were collected. The total cellular protein in the extracts was quantified, and equivalent amounts from each sample were subjected to western blot analysis for the detection of cellular PKR, phosphorylated-PKR (PKR-p), eIF2α, and phosphorylated eIF2α (eIF2α-p), as well as the expression of viral 3CD and 3C proteins. eIF2α expression was detected as a protein loading control. An asterisk marks non-specific bands. A representative result from three independent experiments is shown. **(B)** Cleavage of PKR after EV-A71 infection. RD cells were infected with EV-A71 at an m.o.i. of 10 or treated with STS for 2, 4, 6, 8, and 10 h. Cell extracts were collected at the indicated times for immunoblotting analysis to detect the cellular PKR and viral 3A protein levels. PARP cleavage was examined as an apoptosis marker. GAPDH expression was detected as a protein loading control. Asterisks indicate non-specific bands. A representative result from at least three reproducible experiments is shown.

We have shown previously that EV-A71 infection induces the phosphorylation of eIF2α by PKR, resulting in a gradual shutoff of translation during infection (Jheng et al., 2010). It is worth noting that PKR is not only phosphorylated but also degraded by EV-A71, consistent with observations during PV infection (Black et al., 1989, 1993; Figure 1A). We determined the activation status of endogenous PKR over the time course of EV-A71 infection by immunoblotting with antibodies against phosphorylated PKR and its downstream substrate eIF2α in human RD cells (Figure 1A). The phosphorylated PKR and eIF2α became detectable at 5 h p.i., whereas viral 3C and 3CD proteins were detected as early as 3 h p.i., suggesting that EV-A71 triggers PKR activation at an early stage of infection. In addition, the PKR levels were decreased after viral infection, which was likely the result of the inhibition of translation mediated by eIF2α phosphorylation, or apoptosis-induced PKR cleavage. Because the reduction in PKR occurred simultaneously with its phosphorylation, we ruled out the possibility of translation inhibition. We treated RD cells with staurosporine (STS) to induce apoptosis to determine the status of PKR (Belmokhtar et al., 2001; Figure 1B). As expected, STS treatment caused apoptosis, as indicated by the cleavage of PARP. This apoptosis caused a smaller decrease in the PKR level than that seen in the EV-A71-infected cells, and produced different cleavage patterns compared to those seen in viral infection. We detected a small cleavage product (about 20-kDa) of PKR at 8 h p.i. Therefore, the reduction in PKR might result from proteolysis induced by EV-A71 infection.

### Viral 3C protease cleaves PKR at a site distinct from that cleaved during apoptosis

**Figure 2:**
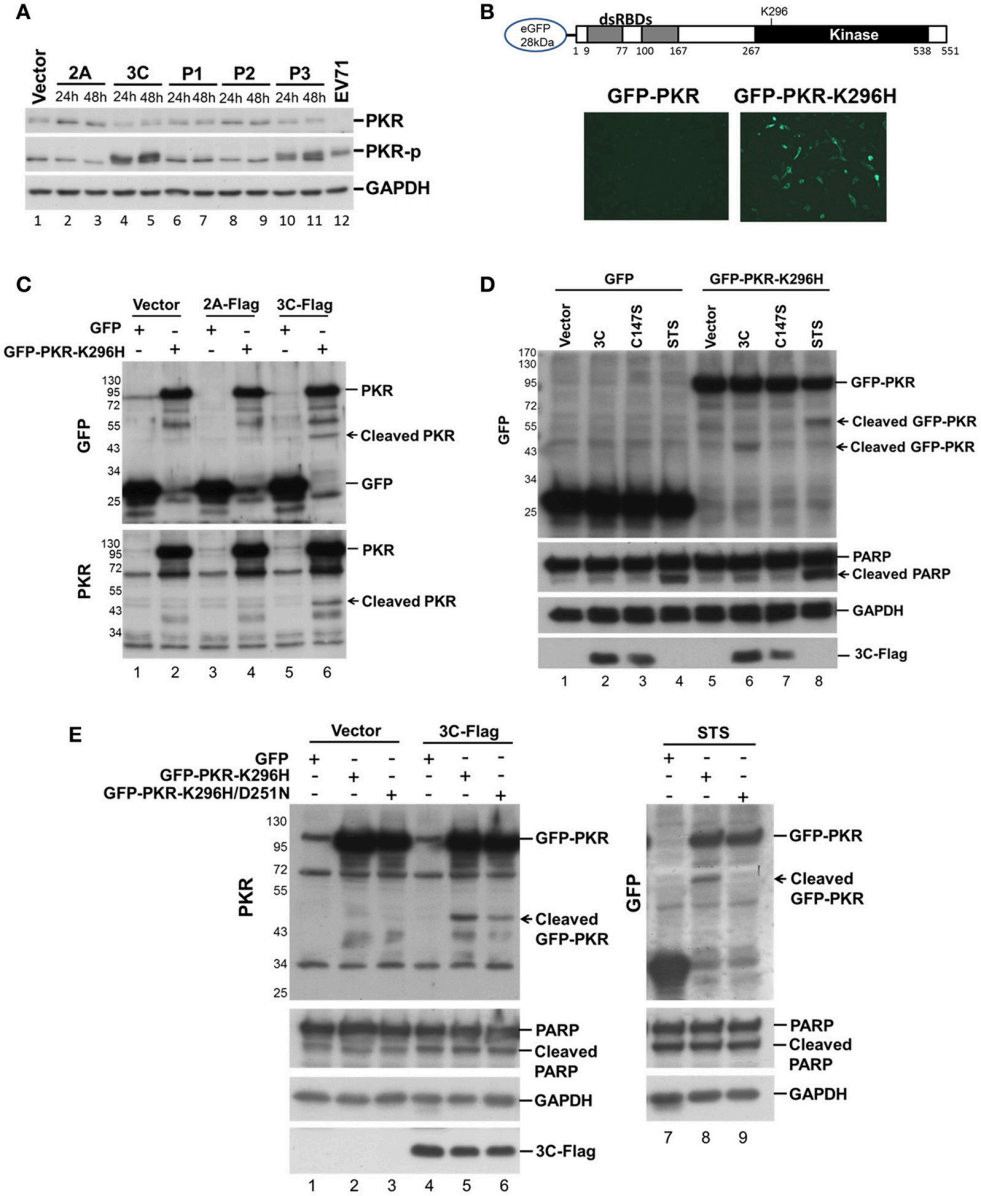
Viral 3C protease is related to the cleavage of recombinant PKR. **(A)** Plasmids expressing viral 2A, 3C, P1, P2, and P3 were transfected into RD cells for 24 and 48 h. Equivalent amounts of protein from the cellular extracts were analyzed by immunoblotting using anti-PKR, PKR-p, and anti-GAPDH specific antibodies. An EV-A71-infected cellular extract served as a positive control. A representative result from three independent experiments is shown. **(B)** Schematic structure of the recombinant PKR, which has an N-terminal-linked GFP to allow monitoring of the cleavage product. RD cells were transfected with GFP-PKR or GFP-PKR-K296H for 24 h and the GFP expression was monitored using an EVOS fluorescence cell imaging system. **(C)** Plasmids expressing GFP-PKR-K296H were transfected into 293T cells for 24 h which were then transfected with the plasmid encoding 2A-Flag or 3C-Flag for another 24 h. Cell extracts were collected for immunoblotting using anti-GFP and anti-PKR antibodies. **(D)** A comparison of the cleavage patterns for 3C- and STS-induced PKR proteolysis. The 293T cells were transfected with GFP or GFP-PKR-K296H for 24 h and were then transfected with either empty vector, 3C-Flag, or C147S for another 24 h, or treated with STS for 8 h. Cell extracts were analyzed by immunoblotting using anti-GFP, anti-PARP, anti-GAPDH, and anti-Flag antibodies. A representative result from at least three reproducible experiments is shown. **(E)** Viral 3C cleaves PKR at a site that differs from that of apoptosis-induced PKR cleavage. The 293T cells were transfected with GFP, GFP-PKR-K296H, or GFP-PKR-K296H/D251N for 24 h, and were then transfected with either vector or 3C-Flag for another 24 h, or treated with STS for 8 h. The cell extracts were analyzed by immunoblotting using anti-PKR, anti-PARP, anti-GAPDH, and anti-Flag antibodies. A representative result from at least three reproducible experiments is shown.

We investigated which viral protein was responsible for the reduction in PKR by individually overexpressing the viral proteases 2A and 3C and the intermediate P1, P2, and P3 fragments in RD cells and then measuring the endogenous PKR levels at 24 and 48 h post-transfection (Figure 2A). Overexpression of viral 3C (lanes 4 and 5, Figure 2A) and the P3 fragment from which 3C is derived (lanes 10 and 11, Figure 2A) caused an increase in phosphorylation and a moderate decrease in PKR, suggesting the possibility that viral 3C protease itself could contribute to PKR degradation. Viral proteases process not only viral polyproteins but also a number of host innate immune proteins (Pathinayake et al., 2015). We hypothesized that the reduction in PKR was caused by viral protease cleavage. To confirm this hypothesis, we generated recombinant GFP-PKR-K296H (a kinase-dead mutant), which could be preferentially expressed in cells relative to wild-type GFP-PKR (Figure 2B). We then used GFP-PKR-K296H to monitor the cleavage pattern. We expressed GFP-PKR-K296H in 293T cells for 24 h and then transfected the cells with 2A-Flag or 3C-Flag for another 24 h. We were able to detect a cleavage product of 45 kDa when PKR was coexpressed with 3C but not when it was coexpressed with 2A or vector control (lane 6 compared with lanes 2 and 4, Figure 2C). These results demonstrate that viral 3C protease but not 2A protease was associated with cleavage of the recombinant PKR.

Because it has been demonstrated that PKR is cleaved by caspase at an early stage of apoptosis (Saelens et al., 2001), and because previous studies have also shown that 3C induces apoptosis when expressed in neuronal cells (Li et al., 2002), the cleavage we observed probably occurs as a result of 3C-induced apoptosis. We further compared the PKR cleavage patterns between 3C coexpression and STS-induced apoptosis, and used coexpression of the 3C catalytic site mutant C147S as a negative control (Figure 2D). The fragment resulting from 3C-induced cleavage of PKR was smaller than that produced by STS-induced cellular apoptosis (60 vs. 45 kDa, compare lanes 6 and 8, Figure 2D), although the apoptosis marker PARP was still cleaved when 3C was expressed. To confirm this finding, we generated a GFP-PKR-K296H/D251N mutant; in which D251, a site known to be cleaved by caspases 3, 7, and 8 during cellular apoptosis, is mutated. The D251N mutation abolished the STS-induced PKRcleavage (lanes 8 and 9, Figure 2E), but not that induced by 3C (lanes 5 and 6, Figure 2E). These results suggest that 3C cleaves PKR at a site that differs from that cleaved during apoptosis.

### Amino acid residues Q188–S189 in PKR are necessary for 3C protease-mediated cleavage

**Figure 3:**
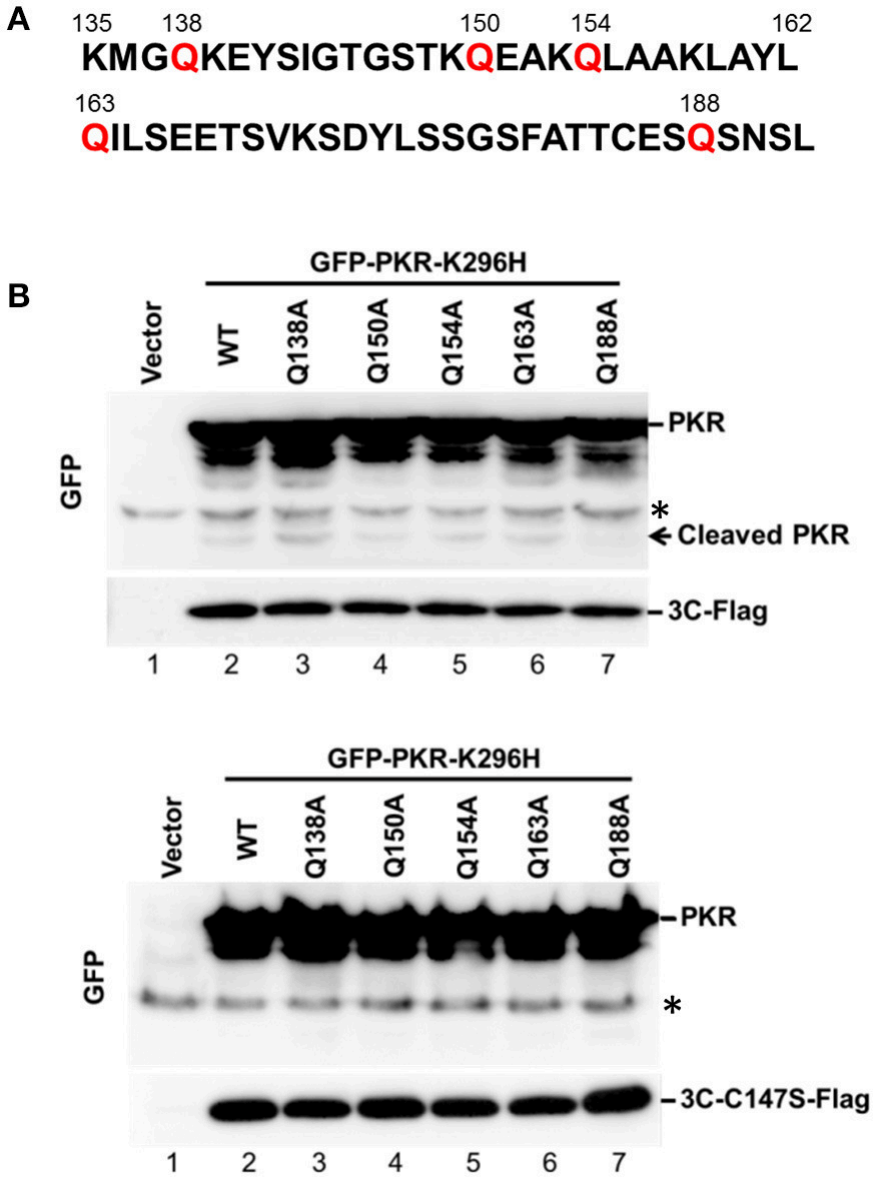
The Q188–S189 pair is the site of PKR cleavage. **(A)** Primary sequences of amino acids 135–192 within PKR. In this region, glutamine residues at sites 138, 150, 154, 163, and 188 were replaced with alanine residues by site-directed mutagenesis. **(B)** The potential 3C cleavage site in PKR. The 293T cells were cotransfected either with plasmids encoding the 3C and GFP-PKR variants (upper panel), or plasmids encoding the 3C mutant C147S and GFP-PKR variants (lower panel). At 24 h after transfection, the cell lysates were subjected to western blot analysis using antibodies against GFP and Flag. A representative result from at least three reproducible experiments is shown. ^*^Denotes non-specific bands.

Because 3C protease-mediated PKR cleavage produces a 20-kDa N-terminal product, we inferred that a cleavage site may exist between amino acids 138 and 188 (Figure 3A). This region contains several glutamine residues and resembles the signature Q-G/Q-S sequence of cleavage sites for other enterovirus 3C proteins. To map the potential 3C-cleavage site of PKR, we constructed a series of mutants in which Q was replaced with A. These mutants were coexpressed with 3C or its mutant C147S in 293T cells, and cell lysates were subjected to western blot analysis. As shown in Figure 3B, Q188A was more refractory to cleavage by 3C than other mutants, suggesting that the Q188–S189 pair is a major cleavage site of 3C within PKR (lane 7, top, Figure 3B). No cleavage of PKR was detected by the 3C-C147S mutant (lower panel of Figure 3B).

### EV-A71 3C physically associates with PKR and induces PKR phosphorylation

**Figure 4:**
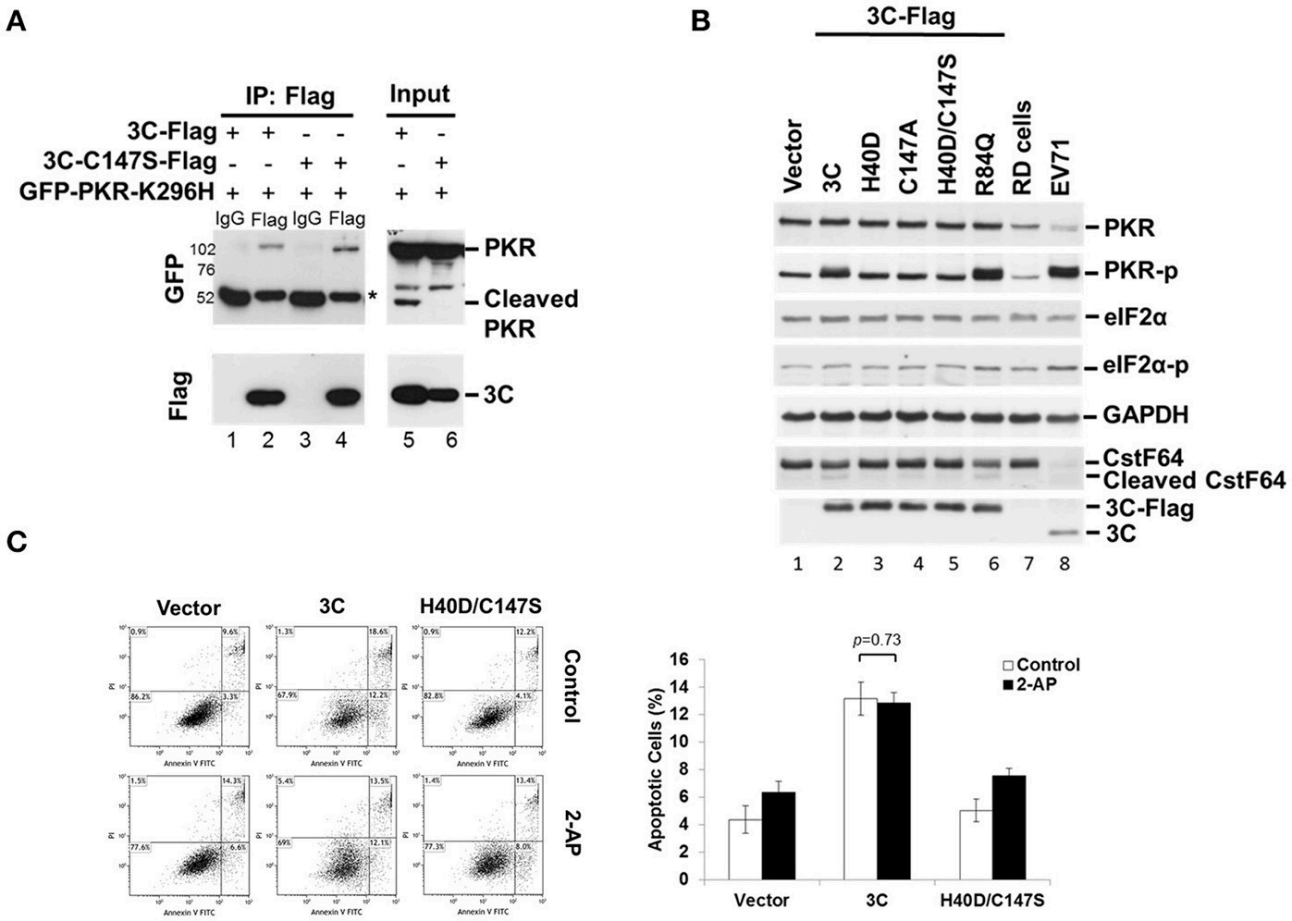
EV-A71 3C associates with PKR and induces PKR phosphorylation. **(A)** The 293T cells were cotransfected with plasmids encoding GFP-PKR-K296H and 3C-Flag, or 3C mutant C147S-Flag, and then harvested at 24 h post-transfection. The cell lysates were immunoprecipitated with antibody against Flag. Samples were then subjected to western blot analysis with detection using anti-GFP and anti-Flag antibodies. ^*^Denotes the heavy chains. **(B)** Mutations of the protease catalytic sites H40 and C147 of 3C caused loss of the PKR phosphorylation activity. RD cells were either transfected with 3C-Flag, H40D, C147S, H40D/C147S, or R84Q, for 24 h or infected with EV-A71/2231 at an m.o.i. of 10 for 8 h. Cellular extracts were collected and immunoblotting was performed for detecting PKR, PKR-p, eIF2α, eIF2α-p, CstF64, Flag, and viral 3C expression levels. CstF64 cleavage was used as a control for 3C catalytic activity. A representative result from three independent experiments is shown. **(C)** The PKR inhibitor 2-AP has no effect on 3C-induced apoptosis. The 3C- or H40D/C147S-transfected 293T cells were incubated with or without 2-AP for 24 h and then analyzed for apoptosis by flow cytometry using Annexin V and PI staining. The values shown in the lower left, lower right, and upper right quadrants of each panel represent the percentage of viable, apoptotic, and dead cells, respectively. Data are the means ± *SD* of values from three independent experiments.

When investigating which viral protein was responsible for PKR reduction, we observed an unexpected phosphorylation of PKR (Figure 2A). Overexpression of viral 3C and the P3 fragment from which 3C is derived strikingly induced PKR phosphorylation, suggesting the possibility that viral 3C protease interacts with PKR and contributes to its phosphorylation. To examine the nature of the 3C–PKR interaction, we carried out immunoprecipitation analysis in 293T cells coexpressing PKR-K296H and 3C or protease-inactive 3C mutant C147S (Figure 4A). The results revealed that both 3C and its C147S variant were specifically associated with PKR (lanes 2 and 4, top panel, Figure 4A), but only the wild-type 3C generated the cleavage product detected in the input (lane 5, top panel, Figure 4A); however, this product was not pulled down by 3C (lane 2 of Figure 4A). We further examined the role of 3C catalytic activity in PKR phosphorylation by examining 3C variants defective in protease activity (H40D, C147S, and double-mutant H40D/C147S; Figure 4B). We used CstF64 as a control for catalytic activity because only wild-type 3C protease could cleave this substrate, although not as robustly as the virus itself (lower panel of Figure 4B). Overexpression of wild-type 3C, the R84Q mutant (defective in RNA binding) of 3C, and EV-A71 infection (lanes 2, 6, and 8, Figure 4B) induced PKR phosphorylation, whereas this capacity was lost in the 3C catalytic-site mutants H40D, C147S, and H40D/C147S (lanes 3, 4, and 5, Figure 4B). These results demonstrated that the protease activity of 3C contributed to the phosphorylation of PKR. Despite this PKR phosphorylation, we noticed that there was no obvious activation of downstream eIF2α. Previous studies have shown that 3C induces apoptosis when expressed in neuronal cells (Li et al., 2002) and that PKR mediates apoptosis (Balachandran et al., 1998). We investigated whether this 3C-induced apoptosis was caused by the activation of PKR. To this end, the PKR inhibitor 2-AP was added to 3C- or H40D/C147S-expressing 293T cells and the status of apoptosis was analyzed by Annexin V/PI staining (Figure 4C). The results showed that 3C, but not H40D/C147S, induced apoptosis in the 293T cells and that this was not abolished by 2-AP treatment. These data, therefore, suggest that although 3C induces PKR phosphorylation, 3C-induced apoptosis is not caused by PKR activation.

### Expression of a catalytically inactive PKR mutant results in increases in viral proteins and virus titer

**Figure 5:**
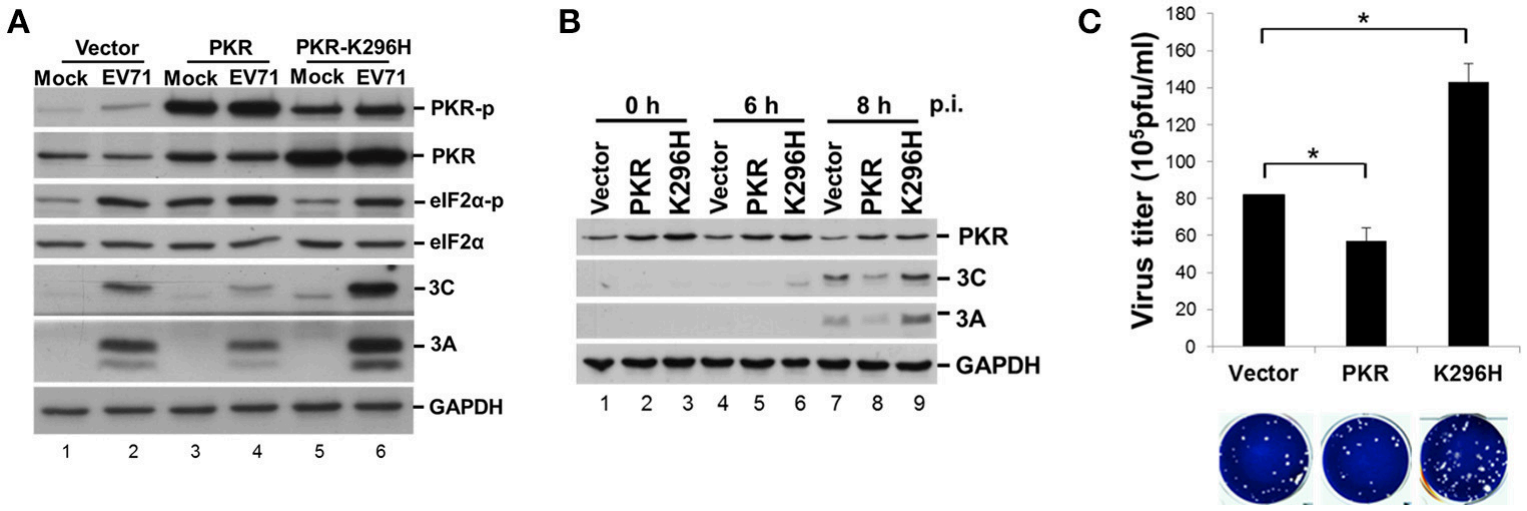
Expression of a PKR kinase-dead mutant results in an increase of viral proteins and virus titer. **(A)** RD cells were transiently expressed with PKR or the K296H mutant for 24 h, and then infected with EV-A71 at an m.o.i. of 10. Immunoblot analysis was performed for detecting the presence and phosphorylation of PKR (anti-PKR, anti-PKR-p) and eIF2α (anti-eIF2α, anti-eIF2α-p), and the expression of viral 3A and 3C proteins. GAPDH expression was used as a protein loading control. A representative result from three independent experiments is shown. **(B)** Stable RD cells expressing vector alone, PKR, or K296H were selected by addition of 3 μg/mL puromycin. Cells were infected with EV-A71 at an m.o.i. of 10 and then the cellular extracts were harvested at 0, 6, and 8 h post-infection. Immunoblot analysis was performed to detect the presence of PKR and viral 3A and 3C proteins. A representative result based on three independent experiments is shown. **(C)** RD cells stably expressing PKR or the K296H mutant were infected with EV-A71 at an m.o.i. of 10 of for 8 h. The RD cells and culture supernatant were harvested for virus titer determination by plaque assay. The results are expressed as the mean ± *SD* (*n* = 3). ^*^p < 0.05.

PKR is an IFN-induced, dsRNA-activated protein kinase that can function as a host antiviral factor. We used a chemical-induced GyrB-PKR model (Ung et al., 2001) to mimic PKR dimerization and activation in cells (Figure S1A). Coumermycin A1 (Cou A) induced dimerization and phosphorylation of wild-type GyrB-PKR, but not the kinase dead mutant GyrB-PKR-K296H (Figure S1B). Dimerization of GyrB-PKR suppressed viral protein synthesis when Cou A was added in the early stage of EV-A71 infection (compare lanes 5 and 6 with lanes 7 and 8, Figure S1B). The suppression effect of PKR dimerization on viral protein expression was abolished in cells expressing the kinase-dead mutant (lanes 11–14, Figure S1B). To further understand its role in EV-A71 infection, we determined the effect on viral infection of a loss of function of PKR. RD cells were transiently transfected with wild-type PKR or the kinase-dead mutant K296H for 24 h and then infected with EV-A71 (Figure 5A). We also generated PKR and K296H stable expression clones in RD cells for the EV-A71 infection experiment (Figure 5B). As expected, PKR overexpression decreased viral protein expression (compare lanes 2 and 4, Figure 5A; lanes 7 and 8, Figure 5B), whereas kinase-dead mutant K296H overexpression increased viral protein accumulation (compare lanes 2 and 6, Figure 5A; lanes 7 and 9, Figure 5B). Consistent with this, the plaque assay results showed that the virus titers were significantly inhibited in cells stably expressing wild-type PKR but were increased in cells stably expressing K296H (Figure 5C). We conclude that the kinase activity of PKR inhibited the replication capability of EV-A71.

### PKR K296H-facilitated EV-A71 replication is abolished by an additional mutation at the dsRNA binding site

**Figure 6:**
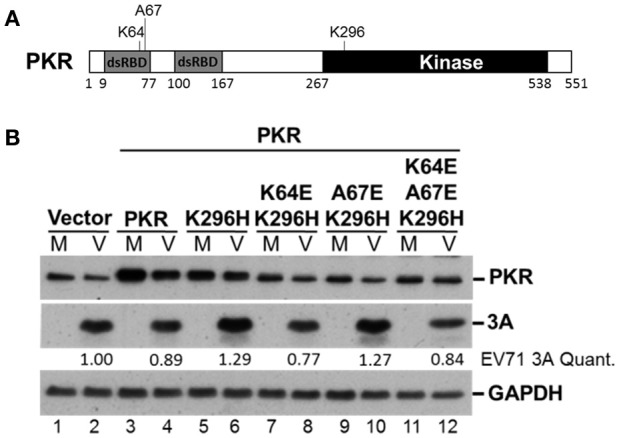
The PKR mutant K296H increases the viral protein accumulation caused by the dsRNA binding domain. **(A)** Schematic representation of the PKR mutants. **(B)** Plasmids encoding vector alone, PKR, K296H, K64E/K296H, A67E/K296H, or K64E/A67E/K296H were transfected into RD cells, which were then selected by the addition of 3 μg/mL puromycin. Stable expressing cells were infected with EV-A71 at an m.o.i of 10 and the cellular extracts were harvested after 8 h. Immunoblot analysis was performed to detect PKR, viral 3A protein, and GAPDH. The relative amount of 3A protein was quantified by densitometry and was first normalized to GAPDH and then to that of the vector control. A representative result based on three independent experiments is shown.

It remains unclear how the catalytically inactive PKR mutant K296H contributes to viral protein accumulation (Figure 5). Because the lysine (K) at amino acid residue 64 contributes to dsRNA binding and the alanine (A) at residue 67 is involved in PKR dimerization (Donnelly et al., 2013), we constructed K64E/K296H, A67E/K296H, and K64E/A67E/K296H mutants (Figure 6A) to clarify the function of PKR in EV-A71 replication. We generated stable expression clones of the wild-type and variant forms of PKR in RD cells, and then infected the cells with EV-A71 at an m.o.i of 10. Immunoblot analysis revealed that the K64E mutant abolished the increase of viral protein accumulation caused by K296H overexpression (compare lanes 6 with 8, and 6 with 12, Figure 6B). In contrast, the A67E mutant had no effect on viral protein expression (compare lanes 6 with 10, Figure 6B). Therefore, the results suggest that the dsRNA-binding activity of the PKR mutant K296H is required for its competition with the antiviral activity of wild-type PKR.

### EV-A71 3C induces PKR cleavage at Q188, generating a short PKR N-terminal fragment that facilitates virus replication

**Figure 7:**
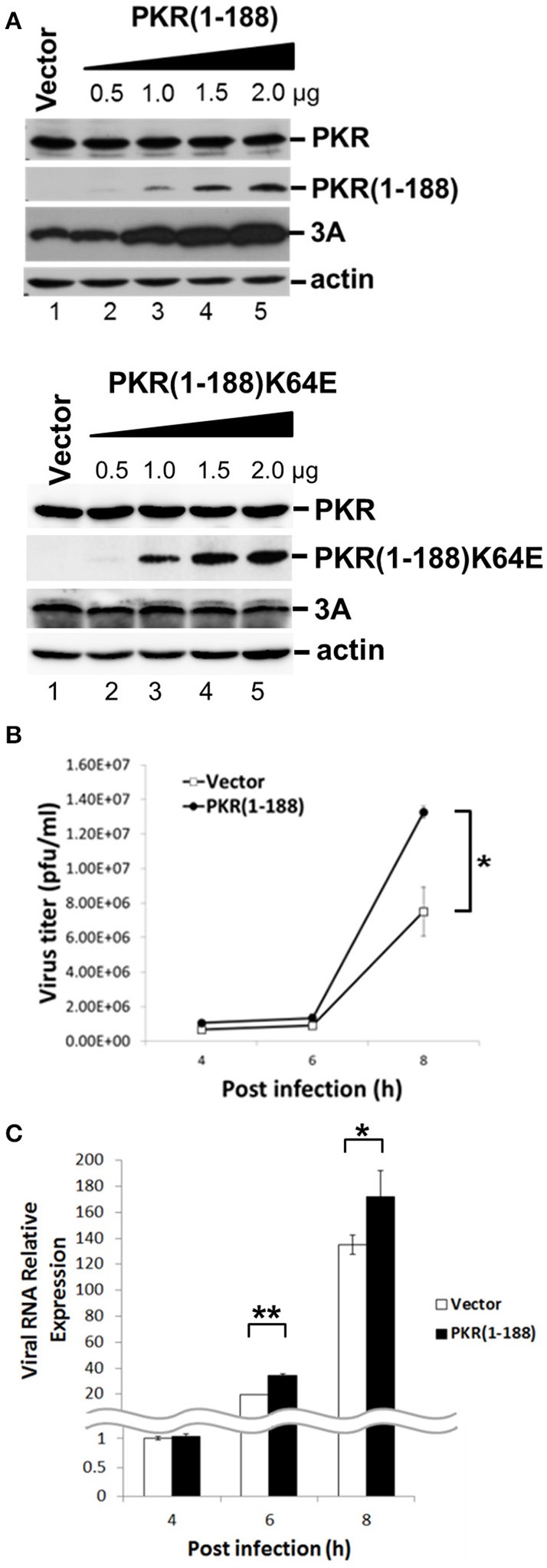
The fragment amino acids 1–188 of PKR facilitates virus replication. **(A)** RD cells were transfected with the vector, increasing concentrations of plasmids encoding the PKR(1–188) or PKR(1–188)K64E fragment. At 48 h after transfection, the cells were infected with EV-A71 at an m.o.i. of 10 for another 8 h. The cell extracts were analyzed by immunoblotting using anti-PKR, anti-3A and anti-actin antibodies. PKR(1–188) was detected with anti-PKR antibody. Actin expression was used as a protein loading control. A representative result from at least three reproducible experiments is shown. **(B,C)** RD cells were transfected with the vector or of plasmids encoding the PKR (1–188) fragment. At 48 h after transfection, the cells were infected with EV-A71 at an m.o.i. of 10 and then the culture supernatant was harvested at 4, 6, and 8 h p.i. for virus titer determination by plaque assay **(B)**; and the total RNA was extracted for intracellular EV-A71 RNA quantification by RT-qPCR **(C)**. GAPDH mRNA quantification was used for normalization. The results are expressed as the mean ± *SD* (*n* = 3). ^*^p < 0.05; ^**^p < 0.01.

Having demonstrated that PKR carrying the kinase-null K296H mutation enhanced viral replication and that this advantage was based on its dsRNA-binding activity, and furthermore that 3C cleaved PKR at Q188 which is outside the dsRNA-binding region, we speculated that the 3C-induced PKR cleavage product may contribute to EV-A71 replication. To determine the function of this small N-terminal PKR fragment, we constructed a plasmid expressing the first 188 amino acid residues of PKR, termed PKR(1–188), and examined its effect on EV-A71 replication (Figure 7). RD cells were transfected with increasing amounts of PKR(1–188) plasmid. Western blotting results showed that PKR(1–188) increased viral 3A protein expression in a dose-dependent manner (top panel, Figure 7A). In contrast, the increase in viral 3A protein expression was eliminated in cells expressing the dsRNA binding mutant PKR(1–188)K64E (lower panel, Figure 7A). To confirm this enhancing effect of PKR(1–188), we analyzed the viral titer (Figure 7B) and viral RNA synthesis (Figure 7C) in a time-course experiment. Consistent with the increase in viral protein expression, viral titer and viral RNA synthesis were augmented in PKR(1–188)-expressing cells compared with vector-control cells. In conclusion, the EV-A71 3C protease cleaved PKR to generate an N-terminal fragment PKR(1–188) that enhanced virus replication.

## Discussion

PKR was recognized as an antiviral protein because of its ability to control translation through phosphorylation of eIF2α, and a number of viruses have strategies designed to counteract its action. PV is the prototype and most intensively studied enterovirus. Previous studies have reported that PKR is highly activated and rapidly degraded in PV-infected cells (Black et al., 1989, 1993). The degradation of PKR in infected cells requires PV gene expression but not PKR catalytic activity. However, PV-encoded proteases 2A, 3C, and 3CD are not directly responsible for the degradation of PKR. Therefore, it was proposed that a cellular protease activated after PV infection was responsible for PKR degradation and that the protease-sensitive site was located within the N-terminal dsRNA-binding domain of PKR. However, we observed PKR cleavage during EV-A71 infection that was not caused by viral infection-induced caspase activation during apoptosis, because it resulted in a different cleavage profile (Figure 1B). Our work demonstrated that in EV-A71, viral protease 3C directly interacts with and cleaves PKR at Q188, proximal to the dsRNA-binding domain of PKR. This difference might be because 3C of EV-A71 has a less strict cleavage specificity than that of PV (Sun et al., 2016). The 3C of PV preferentially cleaves polypeptides with QG junctions, whereas previous studies identified that the 3C of EV-A71 cleaves at a QS site that has an aliphatic amino acid as the fourth amino acid residue preceding the cleavage site (also known as the P4 position) (Lei et al., 2011, 2013, 2014). Our results are consistent with this finding and show that the Q188–S189 pair in PKR, which has an aliphatic leucine residue at the P4 position, represents the signature of the EV-A71 3C cleavage site.

The formation of stress granules (SGs) is a typical response when cells are challenged by viral invasion. Phosphorylation of eIF2α is the major pathway leading to SG formation (Anderson and Kedersha, 2002). PKR is one of the four known cellular eIF2α kinases responsible for translation inhibition during cellular stress, and a recent report indicated that PKR is recruited to the SG complex (Reineke et al., 2015). We also showed that EV-A71 infection induced and modulated endoplasmic reticulum stress for viral replication by inducing PKR-dependent cytosolic accumulation of an endoplasmic reticulum chaperone protein, 78 kDa glucose-regulated protein/binding immunoglobulin protein (GRP78/BiP) (Jheng et al., 2010, 2016). Thus, PKR is a central molecule at the crossroads of innate immune and cellular stress responses because of its capacity to sense dsRNA in virus-infected cells. It has been reported that EV-A71 3C antagonizes the innate IFN response through cleavage of TRIF, IRF7, and IRF9 (Hung et al., 2011; Lei et al., 2011, 2013). The 3C proteins of encephalomyocarditis virus and PV cleave Ras GTPase-activating protein-binding protein 1, a component of SGs, to escape the antiviral responses of SG assembly (White et al., 2007; Ng et al., 2013). Our experiments demonstrate for the first time that PKR is a target of 3C in a strategy to counteract the host antiviral defense.

**Figure 8:**
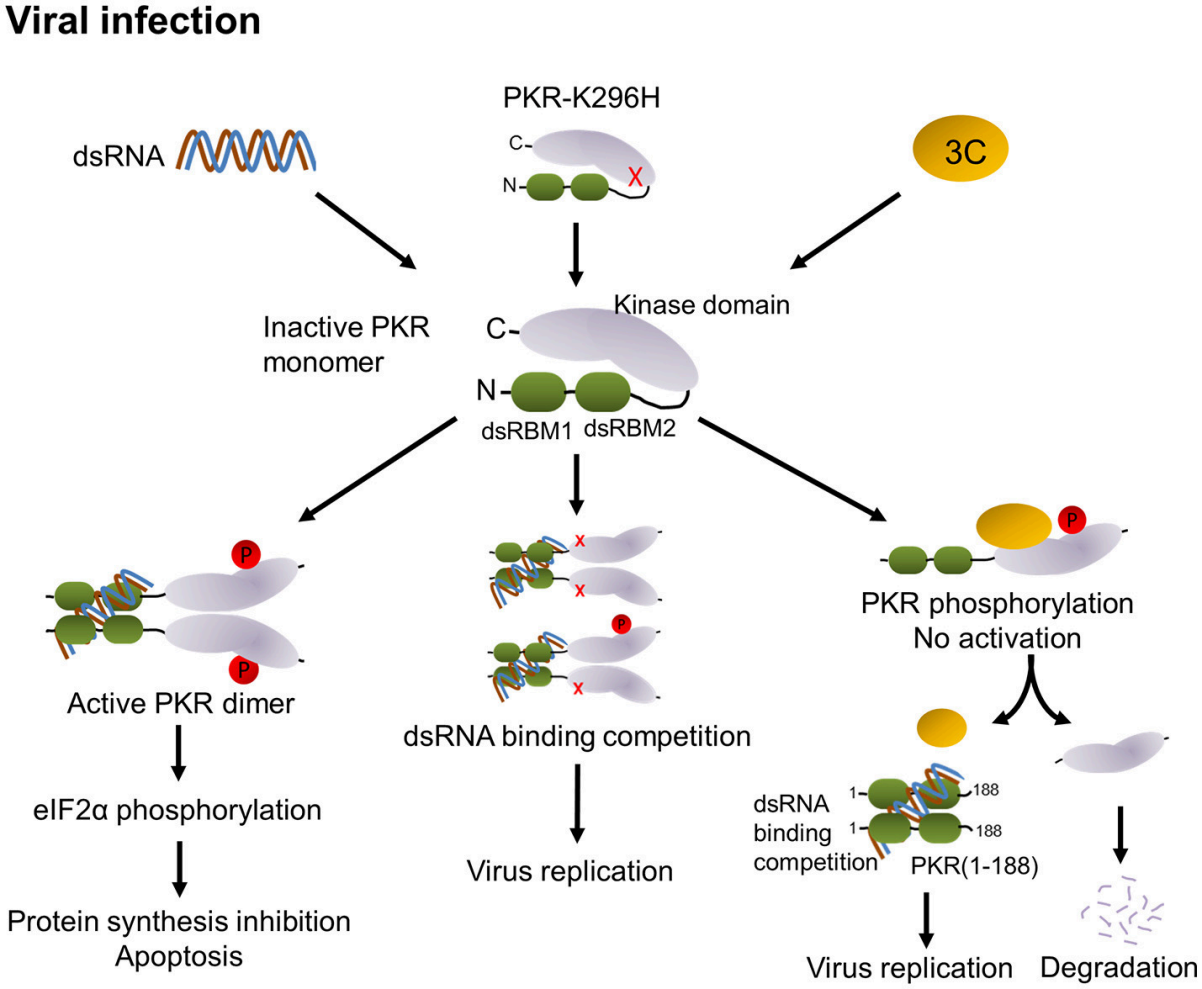
Proposed model of modulation of PKR function by EV-A71 3C protease. PKR consists of two dsRNA-binding motifs (dsRBM1 + dsRBM2 in green) and the C-terminal kinase domain (gray). In general, binding of viral dsRNA leads to dimerization and autophosphorylation of PKR. Active PKR subsequently phosphorylates its substrate eIF2α, which results in translation inhibition and apoptosis (left). Overexpression of the PKR-K296H mutant (cartoon molecule with a red x) competes with endogenous PKR for dsRNA binding to attenuate PKR activation (middle). In EV-A71 infection, 3C interacts with PKR, which may block its dimerization. Then, 3C cleaves PKR to release dsRNA-binding motifs, which may compete with PKR for the recognition of dsRNA, thereby attenuating PKR activation and increasing viral replication (right).

PKR is activated by dsRNA-mediated dimerization. Binding of dsRNA induces a conformational change that leads to the release of the dsRNA-binding domain from the kinase domain and induces dimerization of PKR via the kinase domain, subsequent PKR autophosphorylation and phosphorylation of eIF2α, and results in the inhibition of protein synthesis followed by apoptosis (see graph in Figure 8). In addition to dsRNA, PKR can be activated by protein-protein interactions (Peters et al., 2001). The PKR activating protein (also known as PACT) can heterodimerize with PKR through its dsRNA-binding domain and activate it in the absence of dsRNA. In our study, we observed that 3C expression in cells induces PKR phosphorylation in the absence of dsRNA stimulation and that this induction requires protease activity. In addition, we have demonstrated that 3C directly interacts with PKR (Figure 4A). This suggests that binding to 3C triggers a conformational change in PKR that subsequently allows its phosphorylation. However, the phosphorylation of PKR induced by 3C did not correlate with subsequent phosphorylation of eIF2α (Figure 4B). This result implies that 3C induces PKR phosphorylation but not dimerization, i.e., that 3C might block PKR dimerization. A previous study reported that the 3C protease activity of EV-A71 triggers caspase-dependent apoptosis in human glioblastoma cells (Li et al., 2002). It should be noted here that phosphorylation of eIF2α represents one of the mechanisms by which PKR triggers apoptosis. Our results show that treatment with PKR inhibitor 2-AP was incapable of inhibiting 3C-induced apoptosis (Figure 4C), suggesting that 3C-induced apoptosis is not induced via PKR activation. This result is consistent with our finding that 3C-induced phosphorylation of PKR was independent of PKR activation. However, our data showed that an inactive 3C mutant still interacted with PKR but hardly induced phosphorylation of PKR. We cannot rule out the possibility that the proteolytic activity of 3C induces other unidentified mechanisms that cause the observed phosphorylation of PKR, or that the 3C mutant might induce a transient phosphorylation that we did not detect. Therefore, we propose that 3C negatively regulates the PKR-eIF2α pathway through interacting with and cleaving PKR (Figure 8).

EV-A71 utilize internal ribosome entry sites (IRES) located in the 5′ untranslated region to translate viral proteins in a cap-independent manner (McMinn, 2002). IRES-mediated translation initiation may require both canonical cellular translation factors and auxiliary factors known as IRES trans-acting factors. eIF2 has been identified as a canonical factor that must prebind the 40S ribosomal subunit. eIF2α delivers an initiator tRNAi^Met^ to the small 40S ribosomal subunit in a guanosine-5′-triphosphate (GTP)-dependent manner. Activated PKR catalyzes the phosphorylation of eIF2α, which blocks its capacity to recycle GTP (Holcik and Sonenberg, 2005). Without GTP recycling, eIF2α becomes unavailable to form the eIF2-GTP-tRNAi^Met^ ternary complex, subsequently causing stalling of mRNA translation initiation, and resulting in the global arrest of both cellular and IRES-dependent viral protein synthesis.

In the current study, overexpression of PKR led to increased eIF2α phosphorylation and decreased viral replication (Figure 5), demonstrating the antiviral function of the PKR–eIF2α pathway. In contrast, overexpression of a catalytically inactive PKR mutant led to increased viral protein accumulation and infectious virus yield. The addition of the PKR/K296H mutant decreased endogenous PKR phosphorylation and caused only a moderate decrease in eIF2α phosphorylation during EV-A71 infection (Figure 5A). We postulated that this phenomenon may be the result of competition with endogenous PKR for either dimerization or dsRNA binding. To directly address this possibility, we generated K64E and A67E substitutions in the dsRNA binding site and dimerization site, respectively, within the inactive PKR K296H mutant (Figure 6). The upregulation of viral protein expression by the PKR K296 mutant was eliminated by the additional mutation K64E but not A67E, reflecting that competition for dsRNA binding, rather than for dimerization, was the major mechanism by which the catalytically inactive PKR mutant facilitated EV-A71 replication.

Interestingly, we also discovered that the PKR cleavage during EV-A71 infection generated a short PKR(1–188) product containing two dsRNA binding domains. Our data raised the possibility that this cleavage product of PKR could have a positive effect on virus infection. Indeed, ectopic expression of PKR(1–188) enhanced viral protein accumulation and EV-A71 replication (Figure 7). We hypothesized that PKR(1–188) could have a protective function, similar to that of influenza NS1 protein (Chien et al., 2004), by binding to and sequestering viral dsRNA molecules. However, the cleavage at the Q188–S189 site appeared to be inefficient because the cleavage products were not easily detectable by western blotting. Nevertheless, our results suggested that small amounts of the cleavage products may be sufficient to facilitate EV-A71 replication.

A previous study showed that the N-terminal 1–265 amino acids of PKR are responsible for the activation of the NF-κB signaling pathway by interacting with the IκB kinase complex, whereas the 1–180 amino acids restricted to the two dsRNA binding domains were unable to stimulate an NF-κB response (Bonnet et al., 2006). The N-terminal cleavage product of PKR by 3C precisely eliminates the mechanism designed to stimulate NF-κB activation. Furthermore, the C-terminal 258–551 amino acid kinase domain of PKR generated by caspase cleavage cooperates to activate PKR (Kalai et al., 2007). This reminds us of the possibility that the C-terminal cleavage product of PKR by 3C may, in turn, activate the remaining PKR. However, when we generated PKR K296H-Flag plasmid carrying a C-terminal Flag tag and expressed this plasmid in RD cells, the C-terminal cleavage product of PKR was undetectable during viral infection (data not shown). These results suggested that the products of PKR cleavage by 3C are inactive in stimulating an antiviral response, supported by the fact that cleavage of PKR by 3C clearly attenuated PKR function.

In summary, our results identified a novel role for EV-A71 3C in suppressing the cellular antiviral molecule PKR. We propose that 3C employs two mechanisms: (i) interacting with PKR to block its dimerization, and (ii) PKR cleavage, to counteract PKR activation (Figure 8). Overall, the fine-tuning of PKR activation in EV-A71-infected cells would dictate the outcomes of EV-A71 infection.

## Author contributions

Conceived and designed the experiments: YC, KL, RK, and JH. Performed the experiments: YC and KL. Analyzed the data: YC, KL, and JH. Wrote the paper and edited the manuscript: YC and JH.

### Conflict of interest statement

The authors declare that the research was conducted in the absence of any commercial or financial relationships that could be construed as a potential conflict of interest. The reviewer C-HL declared a shared affiliation, though no other collaboration, with the authors to the handling Editor, who ensured that the process nevertheless met the standards of a fair and objective review.
