# Supplementary figures and images for: dsRNA Binding Domain of PKR Is Proteolytically Released by Enterovirus A71 to Facilitate Viral Replication
Source: Front Cell Infect Microbiol. 2017 Jun 28;7:284. doi: 10.3389/fcimb.2017.00284 (PMC5487429; doi:10.3389/fcimb.2017.00284)

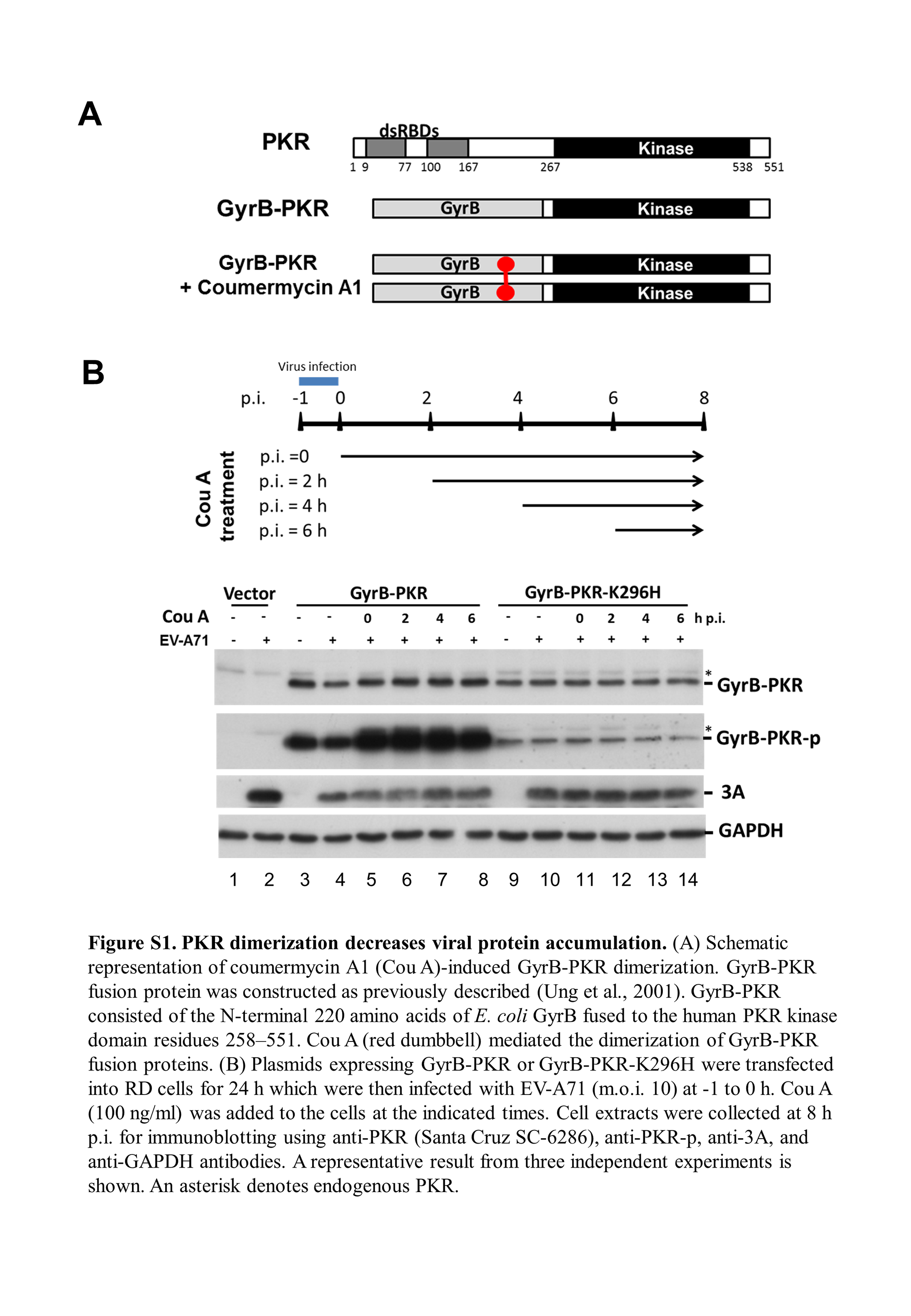

Supplement: Supplementary file 1 [file Image1.TIF]
